# The Bioelectrics of Immortality and Mortality in Cold-Sensitive *Hydra oligactis*

**DOI:** 10.1089/bioe.2025.0002

**Published:** 2025-09-12

**Authors:** Stefania E. Kapsetaki, Angelina Pimkina, Patrick McMillen, Parande Tayyebi, Patrick Erickson, Michael Levin

**Affiliations:** ^1^ Department of Biology, Tufts University, Medford, Massachusetts, USA.; ^2^ Department of Biomedical Engineering, Tufts University, Medford, Massachusetts, USA.; ^3^ Allen Discovery Center at Tufts University, Medford, Massachusetts, USA.; ^4^ Wyss Institute for Biologically Inspired Engineering, Harvard University, Boston, Massachusetts, USA.

**Keywords:** bioelectric map, bioelectric atlas, cnidaria, hydrozoa, bioelectric pattern, membrane voltage, *V*_mem_, resting potential

## Abstract

**Introduction:**

Bioelectric properties of cells are an important aspect of development, regeneration, and cancer. Because of their relevance to the establishment and maintenance of tissue form and function, bioelectric patterns have been hypothesized to have a role in aging. However, no data on bioelectric patterns of the whole body of young and old individuals have been available.

**Methods:**

We observed and quantified the bioelectrics of whole-body immortal (growing at 22°C) and aging mortal (growing at 10°C) cold-sensitive *Hydra oligactis*.

**Results:**

We found that the membrane-voltage-sensitive dyes FluoVolt and VF2.1.Cl can be used to reveal large-scale patterns of cellular membrane resting voltage potentials in hydra. The consensus whole-body bioelectric atlas of immortal hydra shows a consistently depolarized foot and occasionally depolarized tentacles. Immortal hydra are, on average, more depolarized and exhibit less sharply defined bioelectric patterns than old mortal hydra. Immortal hydra have a sharper foot:central body ratio than old mortal hydra.

**Conclusions:**

These data establish hydra as the first model system in which whole-body bioelectric imaging can be performed; the different bioelectric patterns of immortal versus old mortal hydra are consistent with a bioelectric component to the aging process and suggest a roadmap for using this model organism in antiaging therapeutic screens involving electroceuticals.

## Introduction

Aging presents as a severe, progressive reduction in functional capacity and quality of life with a 100% penetrance.^1,2^ It also presents fascinating fundamental questions in evolutionary, cell, and developmental biology with respect to the algorithms implemented by living tissues to maintain form and function against disorder.^3–12^ Numerous theories of the underlying molecular causes of aging have been proposed.^13–21^ One set of approaches focuses on the progressive degradation of developmental information—cues that allow tissues to maintain a complex morphology for decades while materials and individual cells move in and out of the body. Since one key aspect of this information structure is maintained in bioelectrical gradients of resting potential across tissues,^22–24^ it has been suggested that degradation of specifically bioelectric information could be a cause of aging.^25,26^

Patterns of cellular resting potential (*V*_mem_) have been measured in a variety of embryonic, regenerative, and oncogenic contexts,^27–31^ due to the known role of developmental bioelectricity in regulating morphogenesis and cancer suppression.^22,23^ However, no data are currently available on large-scale bioelectric profiles during aging *in vivo* in any model species. This is a rate-limiting step in the development of electroceuticals for longevity. Moreover, additional bioelectricity-relevant approaches involve applying specific electromagnetic fields and pulsed electromagnetic fields to slow down aging,^32–34^ performing transcranial direct current stimulation and transcranial magnetic stimulation to improve the declining cognitive functions,^35,36^ regulating the body’s ion channels with morphoceuticals^25,37^ or gero-electroceuticals,^38^ bioelectrically controlling stem cells such as their ability to differentiate or continue regenerating,^39^ and regulating the restoration of the body’s aging bioelectrics with implantable and wearable bioelectronic devices.^40^ Evaluating, improving, and extending these pioneering approaches require a better understanding of the bioelectric pattern of young versus old individuals. Thus, we sought to establish a dataset on whole-body bioelectric imaging in a model system that is relevant for aging research.^41–43^

Hydra are relatively small freshwater invertebrates. They have endodermal and ectodermal tissues and can reproduce both sexually and asexually.^44^ They have been used as a model organism in many studies^45^ focusing on morphogenesis,^46,47^ environmental ecotoxicology,^48,49^ bacteriology,^50^ regeneration,^41,51,52^ biomechanics,^53^ evolution of multicellularity,^54^ morphogenesis and physiology,^55,56^ transplantation biology,^45^ tumor biology,^57–59^ whole-body neural and muscle activity via calcium imaging,^60–63^ apoptosis,^43,64^ and aging.^42,65^

Their body is transparent, enabling membrane voltage (*V*_mem_)-sensitive dyes^66–68^ to easily penetrate and reveal cellular membrane voltage of the whole organism. At 22°C, cold-sensitive (CS) *Hydra oligactis* are immortal. However, at 10°C, CS *H. oligactis* begin to switch to sexual reproduction and produce ovaries and testes within 3–4 weeks.^69,70^ Within 30 days at 10°, CS *H. oligactis* undergo aging^71^ and eventually die by day 150.^71^ It is known that 80% of the human aging genes from the Human Ageing Genomic Resources (http://genomics.senescence.info) are conserved in hydra.^42^ Aging CS *H. oligactis* are less able to grab prey, perform spontaneous contractions, transfer food to their gut, and have fewer sperm and nurse cells after 2 months, fewer numbers of interstitial stem cells, and fewer epithelial cells.^71^ This makes CS *H. oligactis* an ideal model system in which to begin to test hypotheses about the role of bioelectrics in the aging process. However, the bioelectrics of whole-body immortal and aging hydra are unknown.

We established an imaging protocol for this species and tested the hypothesis that bioelectric patterns (nonhomogeneous spatial distributions of *V*_mem_) would exist and would change with aging, especially becoming less crisp and more diffuse (degradation of prepattern as observed during some birth defects^72^). Moreover, we tested whether aging CS *H. oligactis* would be more or less hyperpolarized than immortal CS *H. oligactis*.

## Methods

### Hydra husbandry and shrimp maintenance

We obtained CS *H. oligactis* from Brigitte Galliot’s Lab. We grew the hydra in media consisting of 0.294 g calcium chloride, 0.07 g magnesium sulfate, 0.08 g sodium bicarbonate (NaHCO_3_), and 0.004 g potassium chloride per 2 L of deionized (DI) water. We stored the hydra in 150 × 15 mm plastic Petri dishes (maximum 50 hydra per dish) in a light:dark 16:8 h cycle incubator at 22°C. We transferred the hydra to a new Petri dish with fresh liquid approximately every 2 weeks. We grew artemia shrimps in a plastic cone oxygenated with an air pump in a light:dark 16:8 h cycle incubator at 22°C. The plastic cone had ∼300 mL of artemia brine shrimp media (25 g sodium chloride and 0.5 g NaHCO_3_ per 1 L of DI) and 0.5 g of artemia brine shrimp egg microcysts. We repeated the artemia brine shrimp growing process from artemia brine shrimp egg microcysts on Tuesdays and Fridays. We fed the hydra approximately one to four artemia brine shrimps per hydra on Mondays, Tuesdays, Thursdays, and Fridays.

### Testing for potential dye toxicity

We tested whether the FluoVolt dye is toxic to the hydra by observing their survival and phenotype after dyeing. Specifically, we collected the hydra from the 22°C incubator. We used five hydra per condition. We imaged the hydra under the Nikon SMZ1500 stereoscope. Then, for the treatment condition, we used the following volumes per hydra: FluoVolt (0.5 μL), power load (12.5 μL), Neuro Background Suppressor 10× (12.5 μL), and hydra media (224.5 μL). For the control, we used the following volumes per hydra: power load (12.5 μL), Neuro Background Suppressor 10× (12.5 μL), and hydra media (225 μL). We placed a hydra in 250 μL of the (control or treatment) solution in a well of a 24-well plate. After 5 min, we washed the well approximately five times with hydra media using a different plastic pipette each time. We imaged the hydra again under the Nikon SMZ1500 stereoscope and then once every day for up to 4 days. During these 4 days, when the hydra were not imaged under the Nikon SMZ1500 stereoscope, they were kept in the 22°C incubator.

### Aging induction

In the beginning of the experiment, after a month since the beginning, after 2 months since the beginning, and after 3 months since the beginning, we imaged four hydra from a culture growing at 22°C and four hydra from a culture growing at 10°C. After 4 months since the beginning of the experiment, we imaged eight hydra from the culture growing at 22°C and eight hydra from the culture growing at 10°C.

### Dyeing and imaging the hydra

We performed the following steps in order to dye and image the hydra. We used fluorescence lifetime imaging microscopy (FLIM) as it allows imaging of the relative membrane voltage in live organisms^73^ and does not rely on dye concentration versus other imaging techniques that rely on image intensity.^68^ On the confocal microscope, we set the chamber temperature at 18°C and 40% air flow. We used the 25× objective (HC Fluotar L Visir 25×/0.95 water) with drops of ultrapure water on the objective. In the Leica Application Suite X (LASX) software, we used the following settings: 1024 × 1024 dimensions; bidirectional mode turned on; line accumulation: 9; speed: 700 Hz; numerical aperture: 0.95; WLL ON at 85% and maximum power; PinholeAiry: 1 AU; laser line (510 nm) and intensity 1.5%; spectral position: 528–617 nm; and gain = 10.

We prepared the dye solution for multiple hydras on the day of imaging using 2 mL Eppendorf tubes. Specifically, we used the following volumes per hydra: FluoVolt (https://www.thermofisher.com/order/catalog/product/F10488) (0.5 μL), power load (https://www.thermofisher.com/order/catalog/product/P10020) (12.5 μL), Neuro Background Suppressor 10× (12.5 μL), and hydra media (224.5 μL). We stored the Eppendorf tubes in the dark at room temperature until using them for each hydra in the next few hours. We collected the hydra from the 22°C incubator and then once finished imaging the 22°C hydra, we imaged the hydra that were growing in the 10°C incubator. We placed a hydra in 250 μL of the dye solution in a well of a 24-well plate. We set a timer for 5 min. We then cut a cover slip on the sides so that it fitted in the middle of an ibidi dish (https://ibidi.com/35-mm-dishes/176-dish-35-mm-high-glass-bottom.html#/29-surface_modification-15h_170_m_5_m_d_263_m_schott_glass_sterilized/30-pcs_box-60_individually_packed). We placed molykote glue on two opposite sides of the ibidi dish (enough glue so that the cover slip could later touch on both sides of the glue). Once the 5 min had passed, we washed the well approximately five times with hydra media using a different plastic pipette each time. We collected the hydra with a plastic pipette and placed it in the middle of the ibidi dish. We placed the cover slip gently on top of the ibidi dish. We took a 10 μL tip with the ibidi dish to the confocal microscope. We placed some additional ultrapure droplets on the confocal 25× objective if needed, so that the liquid touched the bottom of the ibidi dish. We started applying pressure on the sides of the cover slip where the molykote glue had been applied until the glue did not visibly move much further toward the center of the ibidi dish. We made sure the molykote glue did not touch the hydra. We placed the ibidi dish on the confocal microscope stage. We switched the line repetitions on the LASX software to 1, went to tiling mode, and clicked on fast live. We focused on the surface of the hydra (which were not completely immotile) by adjusting the Z plane. We clicked on spiral and identified the whole body of the hydra. We stopped the spiral. Using the polygon tool, we selected the region of interest (the whole hydra). We turned the line repetitions to 9, clicked start, and on the image that was being processed, we clicked on the “Pixel binding” option to convert it to 2. For every image, we clicked on “fit,” then “FLIM image fit,” and used count threshold = 1. The maximum time that the hydra was being imaged under the confocal was 5 min. The pixel dwell time (i.e., how long the laser was on a single pixel) was 0.4–0.6 μs. If each micrometer is approximately 2 pixels, and the typical size of a hydra cell is 10–50 μm, then a cell would typically be imaged within ∼8–40 μs. It typically takes milliseconds for a neural or muscle cell to change its membrane potential and seconds (or minutes) for typical nonneural cells. Thus, a cell would be unlikely to have typically changed its membrane potential by the time the whole cell was being imaged. However, it might have changed its membrane potential by the time the whole frame of the tissue region was being imaged. Completion of a 1024 × 1024 pixel frame takes a few hundred milliseconds. Therefore, if it takes a few milliseconds for a cell to change its membrane potential, a cell might have changed its membrane potential by the time the entire frame has been completed. We then continued dyeing and imaging the next hydra.

### Image analysis of the aging experiment

We obtained the mean lifetime intensity and brightness whole-body images of each FluoVolt-dyed hydra from the LASX software. We transferred these two images of each hydra on ImageJ. We also transferred and ran the following file (Supplementary Data S1) on ImageJ. Downloading this file (Supplementary Data S2) is also required. We then transferred and ran this file (Supplementary Data S3) on ImageJ. In the case of the last file, we manually drew a line surrounding the hydra with the polygon tool. We used the rotation tool to rotate the hydra so that its anterior side was facing left and its posterior side was facing right. We then transferred and ran the following code on ImageJ (Supplementary Data S4). The output values from ImageJ were mean lifetime intensity values across the whole body of the hydra. We transferred these values to Excel, calculated the mean value of the mean lifetime intensity, and the first derivative of these values along the body of the hydra. We also measured the absolute value of the first derivative and the average of the absolute value of the first derivative. The output values from ImageJ also provided values of the x-axis (1 until the total length of the hydra). In order to normalize to the varied length of each hydra, we multiplied each position by 100/(the last × value of the hydra). For example, the last region of the hydra was the 100% region.

To compare the central region and foot region of the hydra, we removed the tentacles by not selecting the tentacles when drawing a line around the hydra on ImageJ. We used the mean lifetime intensity data from the output of this ImageJ analysis and we divided the mean lifetime intensity of the last four regions of each hydra (∼83–100% of their body length) by the mean lifetime intensity of the region that was closest to 50% (of the body length of the hydra).

To measure bioelectric sharpness (Moran’s *I*) of the whole body of the hydra, we transferred the mean lifetime intensity image of the hydra to ImageJ. We adjusted the brightness and contrast so that we could observe the edges of the hydra without applying those settings. Then we ran the following .lut file (Supplementary Data S2). Then, for each image, we cropped the region of interest (whole body of the hydra) using the polygon tool and ran the following macros (Supplementary Data S3). Then, we reset the brightness and contrast settings. We saved the image on the desktop as a TIFF format file. We then ran these two scripts (Supplementary Data S5 and S6) in Idle.^74^

### Consensus bioelectric map of the hydra

To make a bioelectric atlas/map of every hydra, we analyzed separately the mean lifetime intensity whole-body data of the immortal hydra versus the mean lifetime intensity whole-body data of the old mortal hydra. We plotted the mean values of mean lifetime intensity of each hydra that was growing for 2 months (four hydra), 3 months (four hydra), and 4 months (eight hydra) at 22°C against the percentage of its body length on the x-axis and the mean values of mean lifetime intensity of each hydra that was growing for 2 months (four hydra), 3 months (four hydra), and 4 months (eight hydra) at 10°C against the percentage of its body length on the x-axis.

### Image analysis of the VF2.0.Cl-dyed versus VF2.1.Cl-dyed versus FluoVolt-dyed hydra

We dyed 3 three-and-a-half-month-old hydra for 5 min with 250 μL of the following solution (1.5 μL of the VF2.1.Cl dye from a 1 mM stock solution,^75^ 37.5 μL of power load, 37.5 μL of Neuro Background Suppressor 10×, and 673.5 μL of hydra media). We dyed 3 three-and-a-half-month-old hydra for 5 min with 250 μL of the following solution (1.5 μL of the VF2.0.Cl dye from a 1 mM stock solution,^75^ 37.5 μL of power load, 37.5 μL of Neuro Background Suppressor 10×, and 673.5 μL of hydra media). We dyed 3 three-and-a-half-month-old hydra for 5 min with 250 μL of the following solution (1.5 μL of the FluoVolt dye, 37.5 μL of power load, 37.5 μL of Neuro Background Suppressor 10×, and 673.5 μL of hydra media). We performed this procedure separately for each hydra. After the 5 min exposure to the dye in a well of a 24-well plate, we washed the well—four to five times with hydra media. We placed molykote glue on two opposite sides of an ibidi dish and moved the hydra with a plastic pipette to the middle of the ibidi dish. We slowly applied pressure on the sides of the cover slip where the molykote glue was, and imaged the hydra under the confocal microscope using the LASX software and the 25× objective. We used the following settings for all three dyes: 1024 × 1024 dimensions; bidirectional mode turned on, line accumulation: 9; speed: 700 Hz; numerical aperture: 0.95; WLL ON at 85% and maximum power; and PinholeAiry: 1 AU. For imaging the FluoVolt-dyed hydra, we used laser line (510 nm) and intensity 1.5%; spectral position: 528–617 nm; and gain = 10, whereas for imaging the VF2.1.Cl- and VF2.0.Cl-dyed hydra, we used laser line (510 nm) and intensity 30%; spectral position: 533–622 nm; and gain = 10. On the images that were being processed, we clicked on the “Pixel binding” option to convert it to 2. Also, for every image we clicked on “fit,” then “FLIM image fit,” and used count threshold = 1.

We obtained the mean lifetime intensity and brightness whole-body images of the VF2.0.Cl-, VF2.1.Cl-dyed, and FluoVolt-dyed hydra from the LASX software. We dragged these images on the ImageJ software. In the brightness image of each hydra, we looked for six neighboring cells in the foot region and drew a circle around them in the mean lifetime intensity image to measure the average mean lifetime intensity of that region. We also found six neighboring cells from the middle region of the brightness image of the hydra with a maximum ±1 difference in brightness intensity. We drew a circle around those cells in the same position in the mean lifetime intensity image in order to measure the average mean lifetime intensity of those cells. We then compared the average mean lifetime intensity values of those two regions across the different conditions (VF2.0.Cl-dyed vs. VF2.1.Cl-dyed vs. FluoVolt-dyed hydra).

### Statistical analyses

We performed all statistical analyses in R version 4.4.0.^76^ When the data followed an approximately normal distribution (*p* value >0.05 in Shapiro’s test^77^) and we were comparing three or more different groups, we used an analysis of variance test. If the data did not follow a normal distribution (*p* value <0.05 in Shapiro’s test^77^) and we were comparing three or more different groups, we performed the Kruskal–Wallis test.^78^ When the data did not follow a normal distribution (*p* value >0.05 in Shapiro’s test^77^) and we were comparing two different groups, we performed the Wilcoxon rank-sum exact test.^79,80^

## Results

### Hydra survive the dyeing protocol with no consistent deformities

In order to test whether the FluoVolt dye that we used is toxic to the hydra, we compared the survival and phenotypes of hydra exposed to FluoVolt + power load + Neuro Background Suppressor 10× + hydra media versus power load + Neuro Background Suppressor 10× + hydra media. We used five hydra per condition and found no death or consistent large-scale visible deformities in the two conditions throughout 4 days after the exposure to the solution (Supplementary Fig. S3 and Fig. S4). One of the hydra that was exposed to the FluoVolt-containing solution had tentacles prior to staining and 1, 2, and 4 days after staining, but did not have clearly visible tentacles 3 days after staining (top row; Supplementary Fig. S4), either because it had no tentacles on that day or because they had contracted and were behind the anterior region of the hydra and thus were not visible in the 2D image. Another hydra that was exposed to the FluoVolt-containing solution had a circular structure in its gastric cavity only on the second day after exposure to the FluoVolt-containing solution, but not prior to exposure, just after exposure, or on the first, third, or fourth day after exposure to the FluoVolt-containing solution (bottom row; Supplementary Fig. S4). It is not clear whether this circular structure formed due to the dye or due to other reasons, as this circular structure did not appear in any of the other replicates or on any of the other days of imaging. These results show that these two solutions are not life-threatening for the hydra and do not cause deformities across replicates throughout 4 days after exposure.

### V_mem_-sensitive dye reveals relative membrane voltage of every cell along the hydra’s body

In order to determine whether we can use immortal and mortal hydra as a model system to see the relative membrane voltage of every cell, we ran an imaging protocol with the *V*_mem_-sensitive dye FluoVolt. We were able to see the relative membrane voltage of every cell in living immortal and mortal hydra (Supplementary Fig. S1). Depending on the hydra’s body contraction or expansion at the exact time point of imaging, some cells appeared more elongated (e.g., anterior-right region of the hydra in Supplementary Fig. S1A), while other cells appeared less elongated (e.g., anterior-right region of the hydra in Supplementary Fig. S1B). Therefore, our dyeing technique can reveal the whole-body bioelectric atlas of immortal and mortal hydra.

### V_mem_-sensitive dyes FluoVolt and VF2.1.Cl show relative membrane voltage and not artifacts

**FIG. 1.**
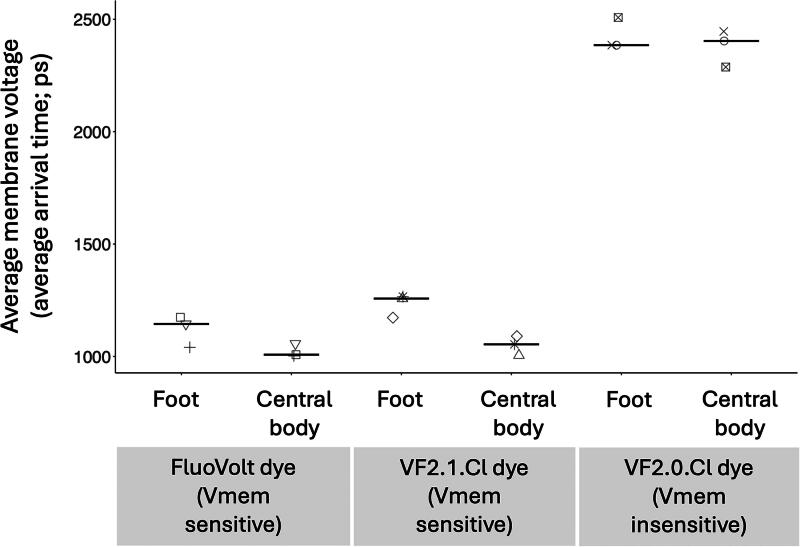
The *V*_mem_-sensitive dyes FluoVolt and VF2.1.Cl show membrane voltage, rather than *V*_mem_-insensitive artifacts. The y-axis shows the mean membrane voltage in the foot or central body regions of three-and-a-half-month-old hydra that have been growing at 10°C, dyed with the FluoVolt dye + power load + Neuro Background Suppressor, VF2.1.Cl dye + power load + Neuro Background Suppressor, or VF2.0.Cl dye + power load + Neuro Background Suppressor. The foot is relatively more depolarized than the middle part of the hydra’s body when using the *V*_mem_-sensitive dyes, but not when using the *V*_mem_-insensitive dye. Same shapes are from the same hydra. There are three different hydra per dye condition. Crossbars show median values. ps, picoseconds.

In order to test whether the *V*_mem_-sensitive dyes FluoVolt and VF2.1.Cl do report relative membrane voltage of hydra cells, we compared the average arrival time of old mortal hydra dyed with FluoVolt, VF2.1.Cl, or the *V*_mem_-insensitive dye VF2.0.Cl. Arrival time refers to the time difference between the excitation of the fluorophore by the laser pulse and the arrival of the emitted photon at the detector.^81^ For example, in the case of *V*_mem_-sensitive dyes, longer arrival time indicates a relatively hyperpolarized cell membrane, whereas shorter arrival time indicates a relatively depolarized cell membrane. In old mortal hydra dyed with FluoVolt (*N* = 3) or VF2.1.Cl (*N* = 3), we saw that the average arrival time of the foot region was longer than the average arrival time of the central body region. However, in old mortal hydra dyed with the *V*_mem_-insensitive dye VF2.0.Cl (*N* = 3), the foot and central body regions had, on average, a similar average arrival time (Fig. 1). The absence of the characteristic voltage pattern when using the closely related but voltage-unresponsive VF2.0.Cl dye suggests that the signals we reported indeed show relative membrane voltage of hydra cells and are unlikely to be due to artifacts unrelated to voltage.

### Immortal hydra have bioelectrically distinct body parts

**FIG. 2.**
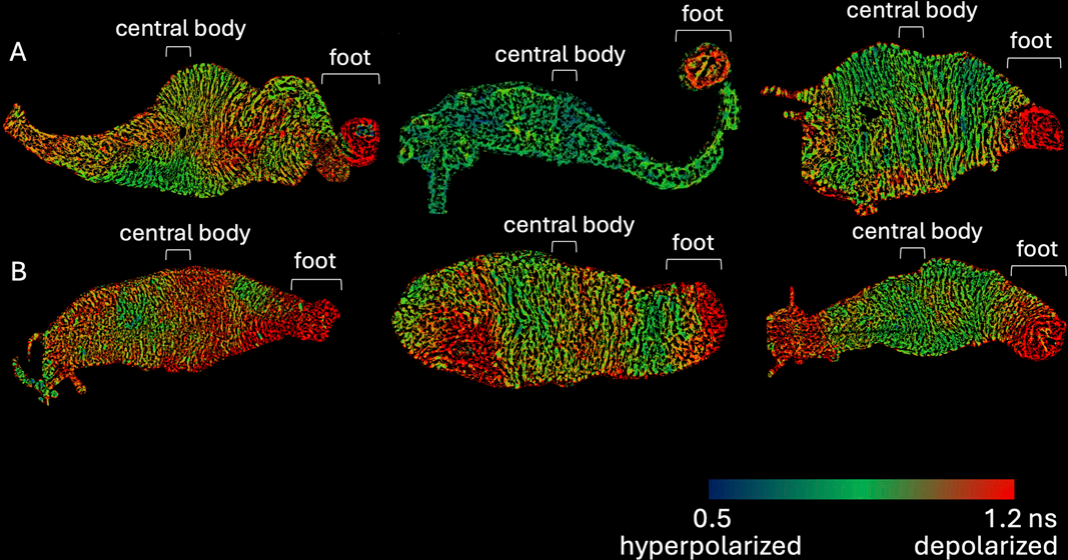
The bioelectric atlas of different individual immortal hydra shows a common feature: a relatively depolarized foot and occasionally depolarized tentacles. Examples of the relative membrane voltage of **(A)** three young hydra at 22°C and **(B)** three young 3-month-old hydra at 22°C. Relative membrane voltage is observed using the FluoVolt dye. Mean lifetime intensity (nanoseconds) ranges from low (blue; hyperpolarized) to high (red; depolarized) values. Images were analyzed in ImageJ software. We aligned the hydra so that their anterior is on the left and their posterior end is on the right.

In order to find the consensus bioelectric atlas of immortal hydra, we imaged hydra growing at 22°C with the *V*_mem_-sensitive dye FluoVolt. We saw that the majority of immortal hydra had a depolarized foot. Some immortal hydra had depolarized tentacles. Other regions of the immortal hydra did not have such consistent membrane voltage among different individuals. For example, the mouth region and central body of the hydra varied in membrane voltage among the different individuals (Fig. 2; *N* = 6). Therefore, the consensus whole-body bioelectric atlas of immortal hydra showed some consistently bioelectrically distinct regions, such as the depolarized foot, and some bioelectrically variable regions, such as the depolarized tentacles, the mouth, and central body.

###  Hydra age at 10 °C

In order to confirm whether hydra age at 10°C, we looked for ovaries or testes on the body of the hydra since gametogenesis in these hydra is a sign of irreversible aging.^70,82^ Hydra that had been growing for 78 days at 10°C had visible ovaries/testes (Supplementary Fig. S2). Therefore, we conclude that in our assays, hydra do indeed age at 10°C.

### Aging hydra have bioelectrically different bodies than immortal hydra

**FIG. 3.**
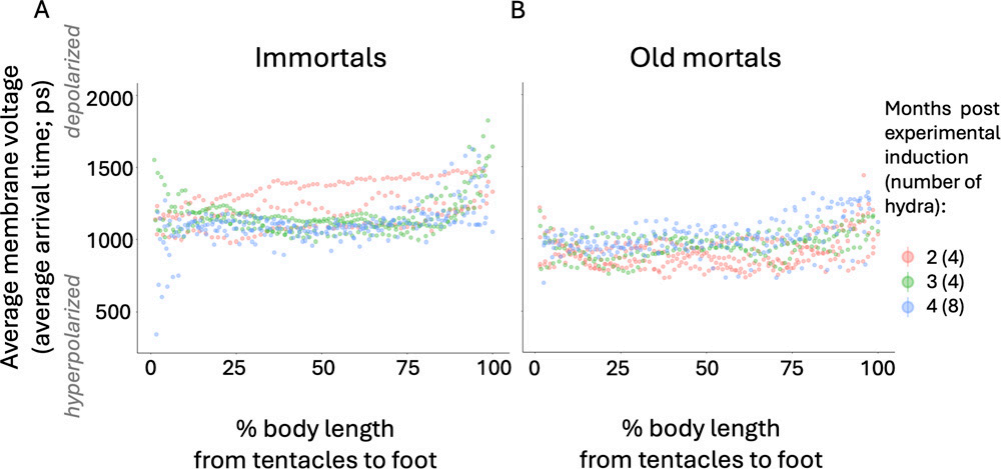
The bioelectric atlas of 16 immortal hydra **(A)** is more depolarized on average, in the foot region, and occasionally in the tentacles in comparison to the bioelectric atlas of 16 old mortal hydra **(B)**. **A** and **B** have the same axes ranges. The mean lifetime intensities of the whole body of immortal hydra were not statistically different (Kruskal–Wallis test: *p* value >0.05) after 2 months (four hydra), 3 months (four hydra), or 4 months (eight hydra). Therefore, we show the mean lifetime intensity (**A**: y-axis) from anterior to posterior (**A**: x-axis) of these 16 different immortal hydra. The mean lifetime intensities of the whole body of old mortal hydra were not statistically different (ANOVA: *p* value >0.05) after 2 months (four hydra), 3 months (four hydra), or 4 months (eight hydra); therefore, **B** shows the means of those 16 old mortal hydra. Therefore, we show the mean lifetime intensity (**B**: y-axis) from anterior to posterior (**B**: x-axis) of these 16 different old mortal hydra. ANOVA, analysis of variance; ps, picoseconds.

**FIG. 4.**
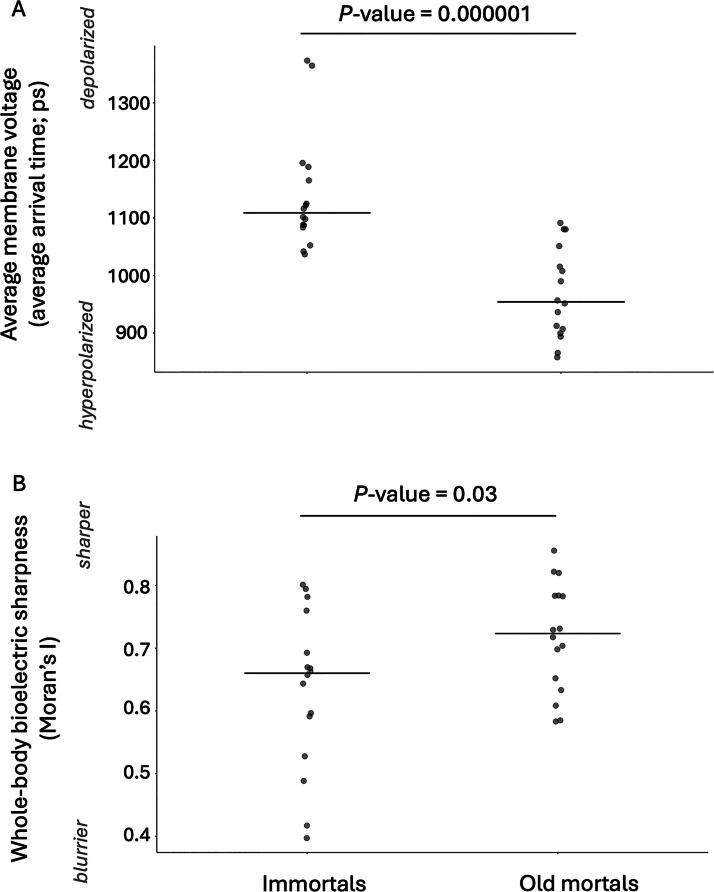
The whole body of 16 old mortal hydra is, on average, more hyperpolarized **(A)** and bioelectrically sharper **(B)** than 16 immortal hydra. **(A)** Average arrival time (ps) of the whole body of old mortal hydra (growing for 2, 3, or 4 months at 10°C; nonstatistically significant difference between the average arrival time values between these three time points; ANOVA test: *p* value >0.05) versus immortal hydra (growing for 2, 3, or 4 months at 22°C; nonstatistically significant difference between the average arrival time values between these three time points; Kruskal–Wallis test: *p* value >0.05). Sharpness **(B)** of the whole body of old mortal hydra (growing for 2, 3, or 4 months at 10°C; nonstatistically significant difference between Moran’s *I* values at these three time points; ANOVA test: *p* value >0.05) versus immortal hydra (growing for 2, 3, or 4 months at 22°C; nonstatistically significant difference between Moran’s *I* values at these three time points; ANOVA test: *p* value >0.05). Each point is a different hydra. The hydra were dyed with the membrane voltage dye: FluoVolt. We only show *p* values that are statistically different (*p* value <0.05). Result of Wilcoxon signed-rank exact test **(A)**: *W* = 242, *p* value = 1.69 × 10^−6^. Result of independent-samples *t* test **(B)**: df = 26.7; *p* value = 0.0374. The crossbars show medians. ANOVA, analysis of variance; ps, picoseconds.

**FIG. 5.**
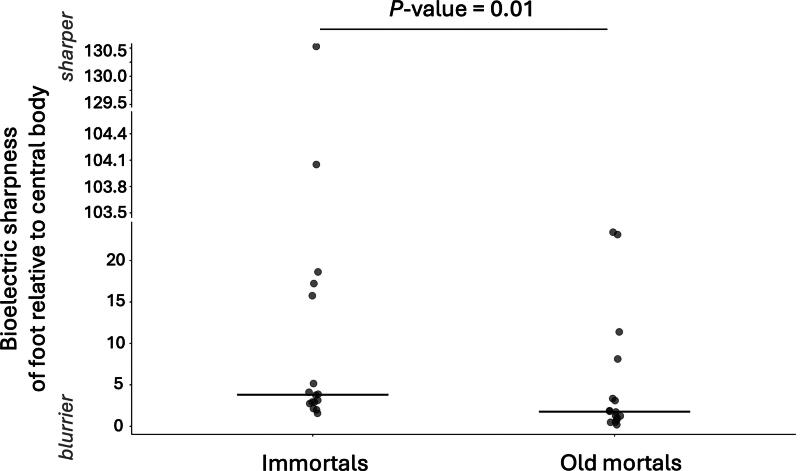
The hydra’s foot (relative to the bioelectric sharpness of its central body region) loses its bioelectric sharpness with age. Each point is a different hydra. Sharpness of the foot relative to the central body of old mortal hydra (growing for 2, 3, or 4 months at 10°C; nonsignificant difference between the *y* values at these time points; Kruskal–Wallis test: *p* value >0.05) versus immortal hydra (growing for 2, 3, or 4 months at 22°C; nonsignificant difference between the *y* values at these time points; Kruskal–Wallis test: *p* value >0.05). The y-axis is the median of the bioelectric sharpness of the foot region (∼83–100% of the hydra, where 100% is the edge of the foot) divided by the mean bioelectric sharpness of the middle region (∼50% position of body length of the hydra). The sharpness of the bioelectric pattern is the absolute value of the first derivative (of the mean lifetime intensity). Result of Wilcoxon rank-sum exact test: *W* = 190, *p* value = 0.01893. After removing the statistically significant outliers (Rosner’s test), the statistical significance remains (two-sample *t* test: *t* = 3.9266; df = 21; *p* value = 0.0007). The crossbars show medians.

In order to test whether aging and immortal hydra have bioelectrically distinct bodies, we grew cultures of hydra in 10°C versus 22°C incubators for 4 months and imaged the relative membrane voltage across the hydras’ bodies using the *V*_mem_-sensitive dye FluoVolt. The technical replicates, that is, two whole-body images from the same individual, varied in mean lifetime intensity (Table 1). Both groups, the technical replicates of the immortal and old mortal hydra, had similar standard deviations (Table 1: 4–46 ps in the case of immortal hydra and 4–47 ps in the case of old mortal hydra). By analyzing one image of the whole body of each hydra, the standard deviation among the biological replicates of Figures 3 and 4A was 101 picoseconds in the case of immortal hydra and 79 picoseconds in the case of old mortal hydra. We saw that the bodies of old mortal hydra, which had been growing for 2, 3, or 4 months at 10°C, were on average more hyperpolarized (*N*_immortal_ = 16, *N*_old mortal_ = 16; Figs. 3 and 4A: *p* < 0.0001) and bioelectrically sharper (*N*_immortal_ = 16, *N*_old mortal_ = 16; Fig. 4B; *p* = 0.03) than immortal hydra, which had been growing for 2, 3, or 4 months at 22°C. The foot-to-central body ratio of old mortal hydra was not as bioelectrically sharp as that of immortal hydra (*N*_immortal_ = 16, *N*_old mortal_ = 16; Fig. 5; *p* = 0.01). Therefore, the bodies of old mortal and immortal hydra have distinct average relative membrane voltage and sharpness of different body regions.

**Table 1.** Technical Replicates from the Same Individual Vary in Their Bioelectrics

Individual	Mean lifetime intensity (picoseconds)		Standard deviation (picoseconds) among the two technical replicates	Time interval (minutes) between the first and second technical replicate
	First technical replicate	Second technical replicate		
First immortal hydra	1009	1074	45.96	3
Second immortal hydra	1066	1125	41.72	3
Third immortal hydra	1061	1040	14.85	1
Fourth immortal hydra	1124	1129	3.54	3
First old mortal hydra	1000	935	45.96	2
Second old mortal hydra	1003	977	18.38	3
Third old mortal hydra	1008	1013	3.54	1
Fourth old mortal hydra	959	893	46.67	2

These are examples of mean lifetime intensity values from technical replicate images of the whole body of four immortal hydra (4 months post-experimental induction at 22°C) and four old mortal hydra (4 months post-experimental induction at 10°C). Higher mean lifetime intensity values indicate relative depolarization, whereas lower mean lifetime intensity values indicate relative hyperpolarization. Longer duration of the hydra under the cover slip, that is, longer time interval between the first and second technical replicate, was not consistently an indication of depolarization or hyperpolarization.

## Discussion

This study provides a consensus whole-body bioelectric atlas of living immortal and mortal hydra. We confirmed that hydra were aging at 10°C since they had formed ovaries/testes within 78 days, an indication that this strain is undergoing irreversible aging.^70,82^

Our results showed that in this model system, the FluoVolt and VF2.1.Cl dyes were showing *V*_mem_ of the plasma membrane and not artifacts, as revealed by the lack of signal from a closely related control dye that is not responsive to membrane potential. This control has been used in cultures of rat hippocampal neurons, human cardiomyocytes, human embryonic kidney cells,^83–85^ and the tails of living frog embryos,^68^ but is not common and should be universally used in voltage dye studies.

Prior efforts have mapped action potentials of the anterior region of the guinea pig’s heart^86,87^; resting potential patterns in bacterial biofilms^88^; central regions of poplar leaves^89^ and stem and leaf of sunflowers *Helianthus annuus*^90^; the optic tectum region of the frog brain^91^; the olfactory bulb of the salamander *Ambystoma tigrinum*^92,93^; faces, brains, and tails of frog embryos^30,68,72^; olfactory bulb of zebrafish^94,95^; olfactory bulb of box turtles *Terrapene triunguis*^96^; an embryonic precontractile chick heart^97^; the pig heart^98^; mouse heart^87,99^; and the primary somatosensory cortex of the mouse brain.^100^ We show for the first time the relative membrane voltage of living cells across the whole body of 38 whole organisms. Certain properties of hydra and technological advances have made this possible. Specifically, hydra has a very thin layer of cells, and thus *V*_mem_-sensitive dyes can easily penetrate these cell layers and be observed under the confocal microscope using FLIM that shows the relative membrane voltage of each cell.

A limitation to note in our data is that we cannot rule out changes in membrane voltage of cells that change their membrane voltage faster (within approximately a few milliseconds^101^) than the completion of the whole-body imaging of a hydra (a few hundred milliseconds: see the section “Methods”). Such signals are unlikely to be critical for developmental bioelectricity because the transduction machinery – and downstream effects on transcriptional changes, epigenetic processes, and cell behavior – all typically take minutes to hours to take place.^102^

With any dye, it is important to consider potential toxicity. We do not believe this was a factor in our study. Regarding potential dye toxicity, any dye toxicity would be present in both hydra conditions, and in the old mortal versus immortal hydra analyses, we are reporting differences in the relative bioelectric pattern of these two conditions that have both been exposed to the dye and washed using the same protocol. The hydra were alive after the brief exposure to the dye, and when they were imaged under the confocal. The laser line used to image the hydra was in the green region (510 nm) and thus relatively less cytotoxic than if a laser line in the blue region was used.^68^ If the dye was toxic, we would expect the entire body of the hydra to be depolarized; however, the hydra’s body was not entirely depolarized. Also, Miller et al. have found that VoltageFluor dyes have almost no capacitance compared with other *V*_mem_ sensors, so they have relatively little effect on *V*_mem_.^75^ Additionally, exposure to the FluoVolt dye did not affect survival of the hydra or consistently change the morphology of the hydra 4 days after exposure to the dye (Supplementary Fig. S3 and Fig. S4)—a much longer time period than the minutes timescale on which we stained and imaged.

One of the key aspects of bioelectric patterns is that in some systems (*Xenopus* embryo, regenerating planaria, chick embryo), their distribution has been shown to serve as an instructive signal for morphogenesis.^30,103–106^ Thus, we were very interested in differences in *V*_mem_ across the hydra that would correspond to distinct anatomical features. We found that the foot of the hydra was more depolarized than other regions of the hydra’s body. The tentacles of the hydra were also relatively depolarized in some individuals. This relative depolarization may relate to the adhesive properties of the foot and secretory properties of the hydra’s tentacles. The hydra’s foot (also called basal disc) secretes granules that contain glycans and/or glycoproteins, allowing the hydra to stick on surfaces.^107^ Although the resting membrane potential of the secretory cells of the foot has not been previously reported, other secretory cells, called nematocytes, in the tentacles of *Hydra vulgaris* are known to require apical plasma membrane depolarization for the secretion of stenoteles.^108^

As with any biological parameter, we observed some variability across animals. The standard deviation in mean lifetime intensity was higher among the biological than among the technical replicates. Specifically, the standard deviation in mean lifetime intensity among the biological replicates of the data shown in Figure 3 and Figure 4A was 101 and 79 picoseconds in the case of the immortal hydra and old mortal hydra, respectively; whereas the standard deviation in mean lifetime intensity among the technical replicates (*N* = 2; Table 1) was 4–46 ps in the case of four immortal hydra and 4–47 ps in the case of four old mortal hydra. Although the standard deviation in mean lifetime intensity of the technical replicates was only measured from two technical replicates, the higher standard deviation in the case of the biological replicates compared to that of the technical replicates indicates that each hydra does not have the same whole-body mean lifetime intensity as other hydra. There tends to be more interindividual variation in the bioelectric pattern of hydra than temporal (1–3 min) variation in the bioelectric pattern of a single individual. In other words, the lower standard deviation in the case of the technical replicates, versus biological replicates, indicates that data within the ∼4–47 ps standard deviation could be considered temporal intraindividual variation (“noise”) and data beyond the ∼47 ps standard deviation could be considered interindividual variation (“signal”). Future work on the functional role of this parameter in hydra biology will be necessary because it is the precision of interpretation of the voltage profile by body systems that would determine the degree of precision that is physiologically important.

One basic bioelectric parameter to compare in living samples is simply the overall average level of polarization. With respect to this, we found a more hyperpolarized bioelectric pattern in the whole body of 2- to 4-month-old mortal than in immortal hydra. Τhe difference cannot be due to temperature per se since we collected all voltage data at the same temperature. What we observed may be due to dynamic changes among many different types of interacting cells. To be more specific, what we found was reasonable for two reasons. First, we would expect the immortal hydra’s foot and tentacles to be relatively depolarized, due to the depolarization required for excretion of substances in secretory cells present in those regions. If this depolarization becomes dysregulated during aging, we would expect old hydra to be, on average, more hyperpolarized than immortal hydra. Second, hyperpolarization of the plasma membrane is known to determine actin fiber compaction in cultures of bovine epithelial cells,^109^ and actin fibers appear disrupted in aging hydra.^71^ Thus, although these two processes happen in very evolutionary distant species, the disrupted actin fibers of aging hydra may be due to cell hyperpolarization.

There seems to be some variety across organisms in terms of the relationship of polarization level to aging. For example, Type II neurons in the bed nucleus of the stria terminalis are more hyperpolarized in aged mice than in young mice.^110^ In rats, old versus young hippocampal CA1 neurons did not have significantly different resting membrane potentials.^111^ Also, there was no significant difference in the resting membrane potential of old versus young pyramidal neurons of the prefrontal cortex of rhesus monkeys.^112^ In female and male mouse bladder cells, the messenger RNA expression of the *hcn1* gene, which encodes the hyperpolarization-activated cyclic nucleotide-gated (HCN) channel, decreases with age.^113^ *H. vulgaris* also has HCN channels (https://www.ncbi.nlm.nih.gov/gene/100208250; https://www.ncbi.nlm.nih.gov/gene/105844959) which are approximately 1 substitution per site, on the protein sequence, different than the human HCN1-4 channels.^114^ If *hnc1* expression is lower and production of HCN1 proteins is decreased in aging hydra, this may explain the potential inability of cells to maintain their depolarized state as they age, that is, our finding that older mortal hydra are more hyperpolarized than immortal hydra.

In addition to average polarization, it is interesting to consider the crispness of the bioelectric pattern itself: the degree to which bioelectric patterns sharply distinguish compartments with different *V*_mem_.^22,115^ In embryos, a number of teratogens that disrupt developmental morphogenesis do so by making the bioelectric prepattern less sharp.^72,116^ For example, in the *Xenopus* brain, this is known to be the case for chemical and genetic teratogens; crucially, brain morphogenesis, gene expression, and even learning rates can be restored despite exposure to nicotine, ethanol, or mutation of the Notch gene by artificially forcing the bioelectric pattern to become sharper (via overexpression of *hcn2*, which encodes an ion channel).^117–119^ We previously suggested the hypothesis that aging could involve a change of the sharpness of the bioelectrical pattern^25^ and indeed *hcn1* expression decreases with age in mice.^113^ We thus quantified here the sharpness of the bioelectrical patterns in immortal and aging hydra. We found a sharper bioelectric pattern in the whole body of old mortal than immortal hydra. However, the foot-to-central body ratio pattern was less sharp in the old mortal hydra. This suggests either that different organisms handle this in different ways, or more likely, that the relationship between crispness of *V*_mem_ pattern and aging is not uniform across body regions.

Our work points to the need for numerous subsequent studies. In parallel with applying antiaging bioelectric treatments (e.g., *hcn2* overexpression) in hydra, it would be useful to understand the mechanisms underpinning the observed whole-body bioelectric atlas of hydra. What ion channels and pumps determine the membrane voltage of every cell? Are there particular groups of cells that have more coordinated/synchronized changes in membrane voltage than other more distant groups of cells in the hydra’s body? How exactly are the known aging-related physiologies of hydra (decreased ability to capture prey, decreased ability to spontaneously contract and transfer food to the gut*,* fewer sperm and nurse cells after 2 months, fewer interstitial stem cells, and fewer epithelial cells^71^) associated with the bioelectric atlas of old mortal hydra? Relative hyperpolarization of the old hydra’s body may also mean a hyperpolarization of the depolarization-activated nematocytes in the hydra’s tentacles, thus contributing to their decreased ability to capture food. However, the associations between the other old physiologies and the old mortal bioelectric atlas are largely unknown. The availability of protocols for generating transgenic hydra^120–123^ suggests the possibility of using optogenetics and ion channel misexpression to understand the role of bioelectrics in hydra morphogenesis and aging. Likewise, it would be interesting to understand the role, if any, of endogenous bioelectrical states and the abilities of external alternating electric fields to induce reversal and continuation of hydra morphogenesis.^124^ Finally, since applied electric fields have been shown to induce reversible changes in hydra morphogenesis,^124,125^ the future study of bioelectrics in various aspects of hydra biology is likely to reveal much of interest.

Identification of the whole-body bioelectric atlas of immortal and aging mortal hydra is a step toward performing similar studies in other model and nonmodel organisms. Studying the bioelectrics of aging in different taxa across the tree of life and the evolution of bioelectric pathways will help better understand the bioelectrics of aging in clinical settings. Examining the bioelectrics of human cells and tissues in both young and elderly populations would be essential in designing best practices for early human clinical trials using morphoceuticals^25^ or gero-electroceuticals,^38^ that is, drugs that target cellular bioelectrics and aim to reverse aging and aging-related diseases. Comparative developmental electrophysiology across widely distributed phyla is a first step in cracking the bioelectric code toward a better understanding of evolutionary physiology and the biomedicine of aging and disease.
