# Supplementary material for: The Bioelectrics of Immortality and Mortality in Cold-Sensitive Hydra oligactis
Source: Bioelectricity. 2025 Sep 12;7(3):166–79. doi: 10.1089/bioe.2025.0002 (PMC12851869; doi:10.1089/bioe.2025.0002)
Supplement: Supplementary file 1 [file bioe.2025.0002_supplementaryfigures1.docx]

**Supplementary Figure 1. Example images showing the relative membrane voltage across the anterior (left)-posterior (right) body of immortal (A: growing at 22°C) and mortal (B: growing at 10°C) cold-sensitive *H. oligactis*.** Mean lifetime intensity (ns) ranges from low (blue; hyperpolarized) to high (red; depolarized) values. We used the FluoVolt V_mem_-sensitive dye to observe the relative membrane voltage. ns: nanoseconds.
