# Supplementary material for: The Bioelectrics of Immortality and Mortality in Cold-Sensitive Hydra oligactis
Source: Bioelectricity. 2025 Sep 12;7(3):166–79. doi: 10.1089/bioe.2025.0002 (PMC12851869; doi:10.1089/bioe.2025.0002)
Supplement: Supplementary file 2 [file bioe.2025.0002_supplementaryfigures2.docx]

**Supplementary Figure 2. Visible ovaries/testes (red arrows) in cold-sensitive *Hydra oligactis* growing for 78 days at 10°C, indicating that this strain ages at 10°C.** Image obtained with a Nikon SMZ1500 stereoscope.
