# Supplementary material for: The Bioelectrics of Immortality and Mortality in Cold-Sensitive Hydra oligactis
Source: Bioelectricity. 2025 Sep 12;7(3):166–79. doi: 10.1089/bioe.2025.0002 (PMC12851869; doi:10.1089/bioe.2025.0002)
Supplement: Supplementary file 3 [file bioe.2025.0002_supplementaryfigures3.docx]

**Supplementary Figure 3**. **Cold-sensitive *Hydra oligactis* survive FluoVolt dye exposure**. Cold-sensitive *Hydra oligactis* growing at 22ºC were treated using the FluoVolt staining protocol described in this paper (bottom row) or mock-treated with staining solution lacking FluoVolt (top row) and observed directly after staining (Day 0) and for 4 subsequent days. 5 hydras were included per condition. One replicate from each condition is shown. The first row shows images of one hydra over time. The second row shows images of a different hydra over time. Images obtained with a Nikon SMZ1500 stereoscope.
