# Supplementary material for: The Bioelectrics of Immortality and Mortality in Cold-Sensitive Hydra oligactis
Source: Bioelectricity. 2025 Sep 12;7(3):166–79. doi: 10.1089/bioe.2025.0002 (PMC12851869; doi:10.1089/bioe.2025.0002)
Supplement: Supplementary file 4 [file bioe.2025.0002_supplementaryfigures4.docx]

**Supplementary Figure 4**. **FluoVolt possibly induces some morphological changes in some hydra.** Images of two cold-sensitive *Hydra oligactis* (one in the top row and another in the bottom row) growing at 22ºC that were treated using the FluoVolt staining protocol described in this paper and observed directly after staining (Day 0) and for 4 subsequent days. In the top hydra, on day 3, the tentacles are not clearly visible. In the bottom hydra, there is a circular structure in the body cavity of the hydra on day 2 after exposure to the dye, but not before or after day 2, until day 4. Images obtained with a Nikon SMZ1500 stereoscope.
